# The analgesic power of pleasant touch in individuals with chronic pain: Recent findings and new insights

**DOI:** 10.3389/fnint.2022.956510

**Published:** 2022-09-13

**Authors:** Martina Fusaro, Rory J. Bufacchi, Valentina Nicolardi, Luca Provenzano

**Affiliations:** ^1^Fondazione Santa Lucia, Rome, Italy; ^2^Italian Institute of Technology, Genova, Italy

**Keywords:** pleasant touch, chronic pain, treatment, immersive virtual reality (IVR), pain modulation

## Abstract

This mini-review covers recent works on the study of pleasant touch in patients with chronic pain (CP) and its potential use as a treatment. While experiments have demonstrated that pleasant touch, through the activation of CT-afferents and the brain regions involved in its affective value, might reduce the unpleasantness and intensity of induced pain, the interaction between pleasant touch and CP remains under-examined. Some experiments show that CP might disrupt the positive aspects of receiving pleasant touch, while in other studies the perception of pleasantness is preserved. Moreover, only a few attempts have been made to test whether touch can have a modulatory effect on CP, but these results also remain inconclusive. Indeed, while one recent study demonstrated that CT-touch can diminish CP after a short stimulation, another study suggested that pleasant touch might not be sufficient. Future studies should further investigate the psychological and neural interplay between pleasant touch and CP. In the conclusion of this mini-review, we propose a new tool we have recently developed using immersive virtual reality (IVR).

## Pleasant touch

The skin is the organ of our body that mediates most of our interactions with the external world (Morrison et al., 2010). Every centimeter of each individual is covered by millions of mechanoreceptors that are activated when interacting with objects or other living beings. Some mechanoreceptors (e.g., those leading to beta, A-Delta and C-fibers), are involved in somatosensation, contributing to the discrimination of surfaces, the detection of materials, or the perception of pain (Treede, 1995; Reinisch and Tschachler, 2005; Woolf and Ma, 2007). Other receptors, which have recently attracted increasing interest, instead contribute to the social and affective aspect of tactile interactions (Gallace and Spence, 2010). These so-called CT-afferents, found in particular in the hairy skin (Olausson et al., 2010), react mainly when stroked at a particular velocity of 3–6 cm/s. Moreover, in order to activate these afferents, the applied force also needs to be controlled. In fact, previous studies have demonstrated that, together with the optimal velocity, a force between 0.04 and 5 millinewton is required (Vallbo et al., 1999; Ackerley et al., 2014a,b; Watkins et al., 2017; Ackerley, 2022) to activate CT-receptors. Lastly, another parameter which might be taken into consideration while caressing is the temperature: touch at skin-temperature seems to be optimal to elicit the firing of CT-fibers while warm and cool touches activate them to a lesser extent (Ackerley, 2022). Importantly, the firing of these CT afferents is coupled with a subjective sensation of pleasantness.

CT-afferents shape our social lives from birth (Cascio et al., 2019; Fini et al., 2022), affecting our interactions with caregivers, friends and romantic partners. Pleasant touch (also called CT-touch)—likely mediated by CT-afferents—confers many other advantages. Neonates, for example, show decreased heart rate (an index of reduced stress) and increased engagement with the caregiver when caressed at a CT-preferred velocity (Fairhurst et al., 2014). Skin-to-skin contact with new-born children (i.e., kangaroo care) is in many countries considered a fundamental practice of maternal care units, as it promotes successful breastfeeding and improves development of preterm babies (Moore et al., 2016). Even in adult life, as postulated by Morrison (2016), pleasant touch can serve as an important stress buffer, as it might promote bodily allostasis by activating some of the key brain nodes involved in stress regulation. Finally, pleasant touch has recently been shown to improve wellbeing during stressful situations such as COVID (Von Mohr et al., 2021), emotional pain (Sahi et al., 2021), and physiological stress (Triscoli et al., 2017).

## Pleasant touch and reduction of pain

It has been demonstrated that pleasant touch can successfully reduce experimentally induced pain (Leknes and Tracey, 2008). Studies in animals highlighted two possible mechanisms behind these pain-reducing effects of CT-afferents effects on pain reduction. The first mechanism is an inhibitory connection between CT inputs and the substantia gelatinosa – which is located in the spinal cord dorsal horn (Lu and Perl, 2003) – and results in an a analgesic effect modulated by the tonic secretions of TAFA4 neurons (Delfini et al., 2013). The second mechanism is related to the increase of oxytocin release during CT activation (Walker et al., 2017a). Furthermore, studies conducted on human participants have highlighted that the analgesic effect experienced by participants may be linked both to a parasympathetic activity (Di Lernia et al., 2018), as indexed by a decreased heart rate during pleasant touches delivered by a romantic partner (as demonstrated by Triscoli et al., 2017), and by a modulation of the endogenous μ-opioid receptor system’s activity (Nummenmaa et al., 2016). More recently, in an extensive review by Meijer et al. (2022), overviewing the neural basis of affective touch and pain, the authors presented a novel model of the interactions between two somatosensory systems. In their model, the authors hypothesized an inhibitory system in which pleasant touch can prevent pain from reaching ascending pathways, blocking cortical processing (and thus, reducing pain perception). Moreover, they also suggest a potential pain modulation through the down regulation of the insula and the anterior cingulate cortex, which are responsible for the subjective experience of pain (Meijer et al., 2022). It is still up for debate whether the reduction of pain arises from bottom-up inhibitory mechanisms described above or from the modulation of the insula and anterior cingulate cortex itself. While the review by Meijer and colleagues mainly focuses on studies on healthy participants and the model developed seems promising, here we review some of the scant studies that investigated the effects of pleasant touch in chronic pain (CP) patients. Reviewing these recent studies, might allow us to understand whether the model proposed could be applied in CP, in the same way as in healthy individuals.

## Pain

The perception of pain, which promotes protection from actual or potential damage (Baliki and Apkarian, 2015), is a complex sensory and emotional experience (International Association for the Study of Pain). Pain can be classified according to its duration: on the one hand, acute pain is a form that is limited in time and extinguished by the resolution of the underlying pathological process (e.g., when the injury heals). On the other hand, CP is a condition in which pain persists well beyond the normal healing time; since determining the end of the healing time of a pathological process can be difficult, pain is considered chronic when it lasts for more than 12 weeks after its onset (International Association for the Study of Pain). Importantly, CP is a common condition that affects more than 30% of the general population worldwide (Cohen et al., 2021).

CP is an umbrella term that comprises several types of pain and conditions. For instance, CP can emerge as a main diagnosis (i.e., primary CP) when it cannot be explained by another chronic condition (ICD-11). In this case, its etiology is unknown and comprises many conditions (e.g., chronic widespread pain, fibromyalgia, and irritable bowel syndrome). Differently, secondary CP is a secondary symptom of a variety of underlying conditions such as cancer (chronic cancer pain), surgery or tissue trauma (postsurgical and post trauma CP; Treede et al., 2015). Another type of CP is Neuropathic CP, which is caused by a lesion or a disease of the somatosensory nervous system (IASP). Lastly, chronic visceral pain originates from internal organs while chronic musculoskeletal pain arises as part of a disease process directly affecting bones, joints, muscles, or soft tissues.

Nevertheless, regardless of its etiology, CP is a serious worldwide health issue, representing a significant social and economic burden (Gaskin and Richard, 2012). Individuals with CP have a crippling disability for most of their existence which deeply affects their quality of life (Campbell et al., 2016). Given that pain is considered a dynamic multifactorial union of biological, psychological, and social factors, it is difficult to build a unique generalized treatment for CP patients. So far, the best practice for treating patients constitutes an individualized, patient-centered, multidisciplinary approach (Chandler et al., 2021).

## Pleasant touch in chronic pain patients

Pain modulations have been extensively studied in healthy individuals (Villemure et al., 2003; Rhudy et al., 2005; Valentini et al., 2017a,b; Nicolardi et al., 2020, 2022) and CP patients (Bushnell et al., 2013; Yarnitsky, 2015). It has been reported that positive emotions and supportive social environments could decrease pain (Che et al., 2018). In this vein, theoretically pleasant touch could be an interesting tool to reduce painful sensations and CP; however, further experimental investigation is still required.

When people experience pain under normal circumstances, gently touching the aching area usually results in physical and emotional relief (Weze et al., 2005). However, this might not hold for patients that suffer from CP, given that their subjective pain perception is altered (Baliki et al., 2006). Indeed, Hashmi et al. (2013) demonstrated that patients with CP—as compared to people with acute pain—showed more activity related to pain in emotion-related circuitry, as opposed to somatosensory areas (Hashmi et al., 2013).

Further evidence comes from Case et al. (2016), who investigated the involvement of opioids in patients with fibromyalgia (a disorder characterized by primary and widespread CP). Opioids are of particular interest because they are responsible for the rewarding nature of pleasant touch. In these patients, the authors observed fewer differences in terms of intensity and pleasantness between slow (at CT-optimal velocity) and fast touch (sub-optimal velocity) than in healthy participants. This suggests a reduced involvement of CT-receptors during the processing of pleasant touch. Moreover, the effects of Naloxone, an opiate-antagonist, differed between patients and healthy participants. While healthy participants reported increased pleasantness, patients reported decreased intensity of touch. Patients with fibromyalgia were also tested in a study by Boehme et al. (2020), which investigated subjective and neural responses to pleasant touch. In this study, the authors demonstrated that patients had intact neural processing for pleasant touch, but that the touch was considered less pleasant than to a control group. This anhedonia to pleasant touch was coupled with dysfunctional evaluative processing, as evidenced by a decreased activity in the right insula during pleasantness ratings and an activation during pain rating.

Another CP condition in which pleasant touch has been investigated is migraine. In a Lapp et al. (2020) study, initially there was no difference in ratings of pleasantness between patients and controls when they were asked to rate a single stroke at different velocities (1, 3, 10 cm/s as optimal-CT velocity vs. 0.3, 30 cm/s as suboptimal velocity). However, in the second part of the study, participants had to rate repeated pleasant touches: here, only the patients with migraine showed a decrease in pleasantness ratings. This was particularly true for patients experiencing tactile allodynia during headaches. This result suggests that the perception of pleasantness might be subclinically altered in CP patients.

Recently, Gossrau et al. (2021) investigated the perception of pleasant touch in patients with postherpetic neuralgia, a pathology characterized by CP. They reported that patients rated the experience of receiving a pleasant touch as less pleasant than a control group did. Similar results were found by Nees et al. (2019), who investigated the perception of pleasant touch in people with chronic back pain. Their results showed that although the brain regions involved in the perception of pleasant touch were the same across groups (patients vs. control), patients with CP showed reduced reactivity and reduced evaluation of the pleasantness of touch. Moreover, their results highlighted the possibility that the pleasantness of touch could be used as marker for problematic affective processing in CP.

In contrast, Parkinson’s disease patients—who are also affected by CP—reported higher pleasantness of pleasant touch than healthy controls, especially when stroked at a velocity of 3cm/s. The authors suggested that dopamine therapy might have an impact in enhancing the rewarding value of touch, turning it into a potential treatment for patients’ pain (Kass-Iliyya et al., 2017).

As demonstrated by the above studies, patients with CP generally show an abnormal perception of pleasant touch, which is diminished in most of studies but preserved in some. However, not enough studies have been conducted to clearly understand which peripheral and central mechanisms influence this altered perception and whether CP patients react differently, depending on the etiology of their pain.

## Pleasant touch as a treatment

The most common form of touch used to reduce pain is massage (see Field, 2019 for a comprehensive review). There is a crucial difference between massage therapy and the type of touch applied in the majority of studies on pleasant touch, as massage therapy probably also activates receptors responsible for the feeling of pressure (in addition to CT-afferents). This makes evidence on massage therapy less comparable to the literature mentioned so far in this review.

Some recent studies have directly investigated whether treatments based on repeated pleasant touch might have an impact on experienced pain. Di Lernia et al. (2020) recently showed that pleasant touch could significantly reduce pain in patients with CP, after a stimulation of only 11 min. The treatment they developed consisted of a controlled stimulation of CT receptors, at a speed of 3 cm/s with a force of 2.5 mN. All patients suffering from various types of CP (i.e., secondary musculoskeletal pain, neuropathic pain, central pain) rated their pain as less severe after undergoing the treatment. This promising evidence highlights the importance of pleasant touch as a novel method to treat CP patients, especially given the consistent effect across different groups of patients.

However, an important concern about applying touch as therapy is when it may evoke unpleasant sensations. Considering that unpleasant evoked sensations (such as tactile allodynia), as reported in the section above, are associated with reduced CT-receptor reactivity and abnormal affective responses in the brain, the analgesic effects of touch might be disrupted in CP patients. In this view, the model proposed by Meijer et al. (2022), which has been designed for healthy individuals, might need to be adapted for patients suffering from CP, taking into consideration the altered top-down and bottom-up mechanisms. In line with this evidence, in two studies Habig et al. (2017) demonstrated that in CP patients, suffering from small fiber neuropathy and complex regional pain syndrome, pleasant touch was ineffective in modulating their experienced pain (2017; 2021). Furthermore, Habig et al. (2017) showed that pleasant touch only modulates the intensity of experienced heat pain for healthy controls; patients with small fiber neuropathy, in contrast, experienced no such pain modulation by touch. However, it must be noted that the impairment of the small fibers might be directly link with a disruption in the activation of CT receptors, thus, patients with this type of neuropathy might respond differently to touch, compared to other CP patients.

In a more recent study (Habig et al., 2021), the authors studied pain perception in people with complex regional pain syndrome. Here, in the sensory assessment, patients reported less pleasantness to touch than healthy participants, regardless of whether the stroking was applied to the affected limb, glabrous or hairy skin, suggesting a disrupted functioning of the CT receptors. Furthermore, a treatment based on repetitive CT-receptor stimulation did not reduce pain intensity in patients. Taken together, these results suggest that the modulation over pain might be too weak to alter CP and that CT-afferents might have lost their ability to mediate the pleasant aspects of touch in the CP patients. It is important to mention that, differently from Di Lernia and colleagues, Habig applied an external painful stimulation and did not investigate the ongoing pain that CP were experiencing. This is a substantial difference that deserves further investigation. In particular, it has to be clarified whether pleasant touch might only play a role in alleviating ongoing pain or can also reduce externally inflicted pain (that is added to the ongoing one). Lastly, it is worth mentioning that Habig et al. (2021) stroked participants with a soft painter’s brush, at a force of 0.8 Newton. Given that the surface area of the brush was not reported, and given the selectivity of CT fibers to specific pressures [described in Vallbo et al. (1999)], the force applied in Habig’s studies might not have been not optimal to activate CT fibres. Further studies are needed to clarify the conditions that result in successful pain reduction: to do so, multiple parameters (such as the ones described in the introduction to obtain a maximum activation of CT receptors) must be controlled.

## Vicarious perception of touch as a perspective

The studies reviewed here provide contrasting results: while pleasant touch reduces pain in healthy participants (among other benefits to wellbeing), in patients with CP it might not be sufficient to reduce pain. Given the altered perception of pleasant touch at peripheral level (for instance due to the exacerbation of allodynia) one possibility could be to investigate whether the affective component of pleasant touch is preserved in CP patients. To do so, it might be useful to separate the visual component of touch from the tactile feedback on the body. Indeed, an interesting corpus of studies have investigated the mechanisms behind the vicarious perception of pleasant touch, namely when people observe pleasant touches on others. These studies highlighted that the ratings of observed touch match the velocity tuning of CT afferents (Walker et al., 2017b): slow pleasant touches were considered more pleasurable compared to fast neutral touches. Moreover, in a different study, when participants were watching videos of others being stroked at CT-optimal vs.-non-optimal speeds, the posterior insula showed a similar response as to directly felt touch (Morrison et al., 2011). This selective tuning of pleasant touch in the insula may allow the recognition of the affective valence of touch even when the touch is only observed. We recently demonstrated that Immersive Virtual Reality (IVR) can also be used to study vicarious sensation of pleasure without actually delivering a stimulus to participants’ real bodies (Fusaro et al., 2016, 2019, 2021; Mello et al., 2021). In a series of studies, we observed that a purely visual virtual touch applied at CT-receptor activating velocity, without any actual touch on participants’ bodies, can elicit sensations of pleasantness that mimic real life interactions. These sensations depend on different factors, such as the appearance of the “touching” avatar or the velocity of observed touch. We also demonstrated that virtual pleasant touch is not only perceived by participants as pleasant and intense (as compared to a neutral stimulus) but can also elicit objective neurophysiological reactions, such as skin conductance responses (Fusaro et al., 2016, 2019, 2021). Interestingly, in a recent study using IVR, Harvie et al. (2022) highlighted that in a patient with complex regional pain syndrome, pain can be evoked by purely observing virtual stimuli without delivering any physical stimulation. After a stage in which the patient underwent an exposure to virtual stimuli, the pain evoked by virtual stimuli was less frequently triggered, thus reducing perceived pain. Unfortunately, this reduction did not occur outside the IVR settings. Nevertheless, this evidence suggests that IVR exposure might be used to develop a successful treatment for CP. If such a treatment is possible, it will hence also be necessary to test the number of repetitions needed to transpose the benefit of IVR outside the experimental settings. Indeed, previous studies have demonstrated that the changes obtained thanks to IVR procedures can also be observed in follow-up sessions when these procedures are reiterated (Banakou et al., 2016; Freeman et al., 2017). Capitalizing on our evidence and on Harvie’s study, we offer two considerations in support of the idea that virtual pleasant touch could be used to study and treat CP. First, virtual pleasant touch prevents the risk of activating allodynia, since no mechanical touch is performed on participants’ bodies. Instead, the pleasantness elicited by the vicarious perception (which has been shown to activate similar brain regions as first-hand pleasure, Morrison et al., 2011) might have a purely positive effect on perceived pain, particularly on the emotional aspects which are altered in CP patients (Hashmi et al., 2013). Second, patients who have developed avoidance to touch (due to evoked unpleasantness) can benefit from the repeated exposure to virtual touch, and consequently become desensitized to it, as demonstrated by the protocol developed by Harvie and colleagues.

To conclude, the interplay between pleasant touch and pain in CP patients needs further investigations to unravel the bottom-up and top-down mechanisms at play. We hypothesized that two possible disruptions might be at play here. CT- touch directly activates emotional key nodes such as the insula. On the one hand, in CP patients this activation might be altered, as suggested by the study of Boehme and colleagues in which they demonstrated intact perception of pleasant stimuli but an alteration of stimulus evaluation in patients with fibromyalgia. Thus, restoring the positive affective value of pleasant touch might be a fundamental first step to develop a successful treatment. On the other hand, as suggested by Habig et al. (2017, 2021), stimulating CT receptors might be insufficient to alter the perception of pain, probably due to the impairment of the system in CP patients. In this case, it might be fundamental to assess the normal functioning of the receptors on a case-by-case basis, and consequently develop individual-oriented treatments.

## Author contributions

MF: conceptualization, funding acquisition, writing—original draft, writing—review and editing. VN and LP: writing—review and editing. RJB: funding acquisition, and writing—review and editing. All authors contributed to the article and approved the submitted version.
